# Plasma biomarkers reflecting high oxidative stress in the prediction of myocardial injury due to anthracycline chemotherapy and the effect of carvedilol: insights from the CECCY Trial

**DOI:** 10.18632/oncotarget.28182

**Published:** 2022-01-25

**Authors:** Mauro Rogério de Barros Wanderley Jr., Mônica Samuel Ávila, Miguel Morita Fernandes-Silva, Fátima das Dores Cruz, Sara Michelly Gonçalves Brandão, Vagner Oliveira Carvalho Rigaud, Ludhmila Abrahão Hajjar, Roberto Kalil Filho, Edécio Cunha-Neto, Edimar Alcides Bocchi, Silvia Moreira Ayub-Ferreira

**Affiliations:** ^1^Heart Failure Department, Heart Institute (InCor) do Hospital das Clínicas da Faculdade de Medicina da Universidade de São Paulo, São Paulo, Brazil; ^2^School of Medicine, Universidade Federal do Paraná, Curitiba, Paraná, Brazil; ^3^Heart Institute (InCor) do Hospital das Clínicas da Faculdade de Medicina da Universidade de São Paulo, São Paulo, Brazil; ^4^Instituto do Câncer do Estado de São Paulo-Universidade de São Paulo, São Paulo, Brazil

**Keywords:** cardiotoxicity, biomarkers, myeloperoxidase, galectin-3, anthracyclines

## Abstract

Background: Anthracycline (ANT) is often used for breast cancer treatment but its clinical use is limited by cardiotoxicity (CTX). CECCY trial demonstrated that the β-blocker carvedilol (CVD) could attenuate myocardial injury secondary to ANT. Mieloperoxydase (MPO) is a biomarker of oxidative stress and galectin-3 (Gal-3) is a biomarker of fibrosis and cardiac remodeling. We evaluated the correlation between MPO and Gal-3 behavior with CTX.

Materials and Methods: A *post hoc* analysis was performed in the patients who were included in the CECCY trial. A total of 192 women had her blood samples stored during the study at –80°C until the time of assay in a single batch. Stored blood samples were obtained at baseline, 3 and 6 months after randomization. We excluded samples from 18 patients because of hemolysis. MPO and Gal-3 were measured using Luminex xMAP technology through MILLIPLEX MAP KIT (Merck Laboratories).

Results: 26 patients (14.9%) had a decrease of at least 10% in LVEF at 6 months after the initiation of chemotherapy. Among these, there was no significant difference in the MPO and Gal-3 when compared to the group without drop in LVEF (*p* = 0.85 for both MPO and Gal-3). Blood levels of MPO [baseline: 13.2 (7.9, 24.8), 3 months: 17.7 (11.1, 31.1), 6 months: 19.2 (11.1, 37.8) ng/mL] and Gal-3 [baseline: 6.3 (5.2, 9.6), 3 months: 12.3 (9.8, 16.0), 6 months: 10.3 (8.2, 13.1) ng/mL] increased after ANT chemotherapy, and the longitudinal changes were similar between the placebo and CVD groups (p for interaction: 0.28 and 0.32, respectively). In an exploratory analysis, as there is no normal cutoff value established for Gal-3 and MPO in the literature, the MPO and Gal-3 results were splited in two groups: above and below median. In the placebo group, women with high (above median) baseline MPO blood levels demonstrated a greater increase in TnI blood levels than those with low baseline MPO blood levels (*p* = 0.041). Compared with placebo, CVD significantly reduced TnI blood levels in women with high MPO blood levels (*p* < 0.001), but did not reduce the TnI levels in women with low baseline MPO blood levels (*p* = 0.97; p for interaction = 0.009). There was no significant interaction between CVD treatment and baseline Gal-3 blood levels (p for interaction = 0.99).

Conclusions: In this subanalysis of the CECCY trial, MPO and Gal-3 biomarkers did not predict the development of CTX. However, MPO blood levels above median was associated with more severe myocardial injury and identified women who were most likely to benefit from carvedilol for primary prevention (NCT01724450).

## INTRODUCTION

Breast cancer is the most common malignancy among women [1]. Despite advances in the survival of patients with cancer, their prognosis remains limited by complications that are frequently related to therapy [2]. Anthracycline (ANT) is a chemotherapeutic drug with high rates of success, being central for breast cancer treatment, but its cardiotoxic effects have limited its clinical use [2, 3].

The incidence of cardiotoxicity with anthracycline is dose-dependent, reaching up to 20% with doses of 550 mg/m^2^ of doxorubicin [4]. Cardiotoxicity (CTX) has been associated with high mortality, and early detection and treatment are critical to improve survival by either preventing cardiac deterioration or increasing the likelihood of cardiac recovery [5].

Previous studies have suggested that serum biomarkers may identify cardiac injury after ANT chemotherapy and precede the decrease in left ventricular ejection fraction (LVEF) [6]. However, other studies failed to demonstrate an association between troponin (Tn) blood levels at baseline and further development of systolic dysfunction after ANT chemotherapy [7–9].

There has been an effort to find others circulating biomarkers that may contribute to predict CTX. Galectin-3 (Gal-3) is a biomarker involved in cell adhesion, fibroblast activation and apoptosis and has been related to cardiac remodeling and fibrosis [10]. Furthermore, recent studies have shown that oxidative stress and reactive oxygen species (ROS) production may have a central role in the mechanism of doxorubicin-mediated cell toxicity [11]. Myeloperoxidase (MPO) is a pro-atherogenic enzyme produced by neutrophils that leads to free radical production and lipid peroxidation and has been related to oxidative stress [12–14]. The behavior of these circulating biomarkers can provide mechanistic insights on the development of ANT CTX and it has a potential role on identifying individuals who are more likely to suffer from cardiac injury.

Therefore, we evaluated the effect of carvedilol on MPO and Gal-3 blood levels among women with breast cancer undergoing ANT chemotherapy that attended CECCY trial. In addition, we investigated whether these biomarkers of oxidative stress are related with the development of ANT-chemotherapy cardiac injury.

## RESULTS

**Figure 1:**
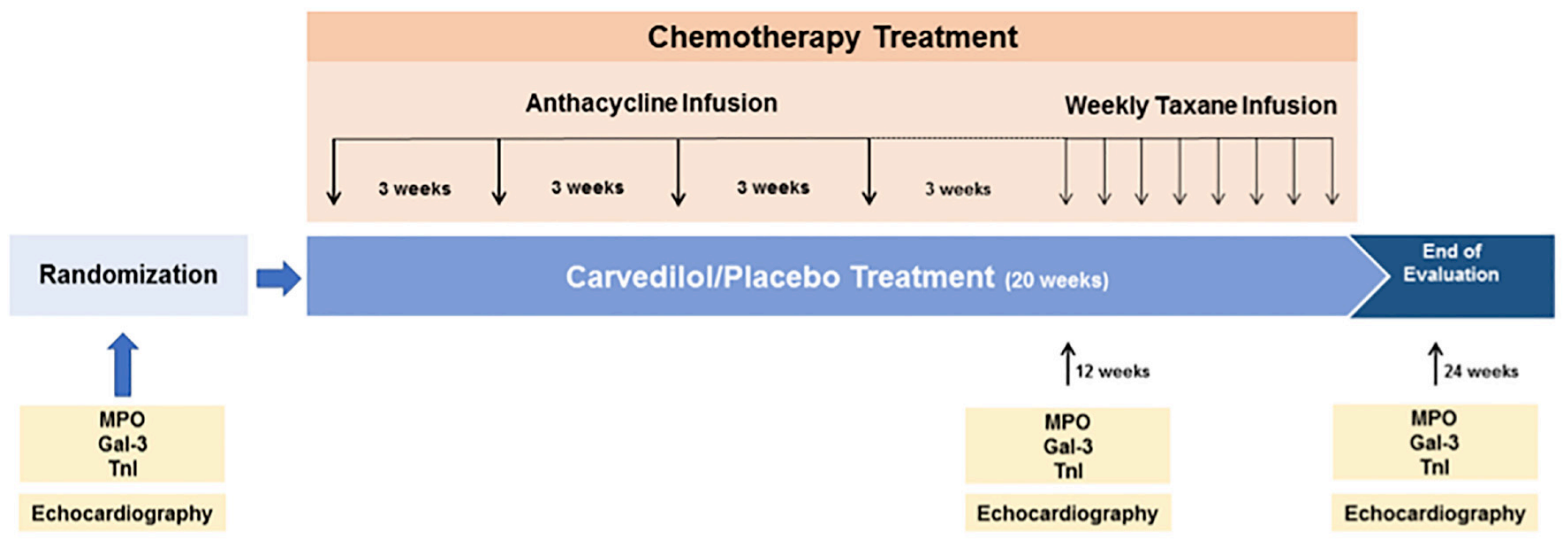
Study design. Subanalysis: 174 patients MPO and Gal-3. Chemotherapy protocol: cyclophosphamide, doxorubicin, paclitaxel. Cumulative ANT (doxorubicin) dose: 240 mg/m^2^.

From April 2013 to January 2017, 200 patients were randomly assigned to receive carvedilol or placebo for the intention-to-treat analysis. Eight patients had no valid randomization. The baseline characteristics of the patients were statistically balanced across groups and were shown in a previous publication [9]. These patients had their blood samples stored at −80°C. A *post hoc* analysis was performed and the blood samples from randomization, 3 and 6 months after the beginning of the study were submitted to MPO and Gal-3 measurements in a single batch. 18 patients had at least two blood samples mislaid. So, 174 patients were included in this study (Figure 1). The baseline characteristics of the population and the number of patients who took carvedilol or placebo are shown in Tables 1 and 2, respectively.

**Table 1:** Baseline characteristics of the patients

	MPO below median	MPO above median	
	*n* = 87	*n* = 87	
Age	49.92 ± 8.81	50.91 ± 10.11	*p* = 0.49
Post-menopause	39 (44.8%)	47 (54.0%)	*p* = 0.23
Therapy	2.47 ± 0.61	2.48 ± 0.50	*p* = 0.89
Hypertension	6 (6.9%)	5 (5.7%)	*p* = 0.76
Diabetes Mellitus	3 (3.4%)	5 (5.7%)	*p* = 0.47
Hypercholesterolemia under statin treatment	4 (4.6%)	6 (7.0%)	*p* = 0.50
Current/past smokers	22 (25.3%)	24 (27.6%)	*p* = 0.73
Systolic blood pressure, mmHg	123.74 ± 15.72	122.10 ± 19.01	*p* = 0.54
Diastolic blood pressure, mmHg	78.02 ± 10.12	78.64 ± 12.50	*p* = 0.72
Heart rate, beats/min	79.55 ± 12.66	83.43 ± 12.28	*p* = 0.042
Maximal tolerated carvedilol/placebo dose, mg/day	12.5 (6.2, 25.0)	25.0 (12.5, 25.0)	*p* = 0.18
Serum troponin I baseline ng/ml	0.0 (0.0, 0.0)	0.0 (0.0, 0.0)	*p* = 0.80
Serum BNP, pg/ml	14.0 (8.0, 25.0)	13.0 (6.0, 21.0)	*p* = 0.48

**Table 2:** Treatment based on MPO value

	Placebo	Carvedilol	Total
MPO below median	43	44	87
MPO above median	43	44	87
Total	86	88	174

### Primary endpoint

During the follow-up, 26 patients (14.9%) had a decrease of at least 10% in LVEF at 6 months after the initiation of chemotherapy. The trajectories of the MPO and Gal-3 blood levels were similar between women with and without substantial drop in LVEF (*p* = 0.85 for both MPO and Gal-3); (Table 3). The rise of biomarkers occurred in both groups, although there was a linear increase in MPO and an increase followed by a decrease in Gal-3.

**Table 3:** Drop in LVEF based on biomarkers measurements

	No drop in LVEF	Drop in LVEF ≥10%	*P* value
	*N* = 148	*N* = 26	
Galectin 3 ng/ml			0.85
Baseline	6.3 (5.2, 10.0)	6.3 (5.0, 8.8)	
12 weeks	12.4 (9.8, 16.1)	11.0 (9.8, 14.2)	
24 weeks	10.3 (7.6, 12.8)	10.4 (8.5, 12.6)	
Myeloperoxidase ng/ml			0.85
Baseline	13.2 (7.5, 26.0)	13.3 (10.8, 18.7)	
12 weeks	18.1 (12.3, 39.1)	14.1 (10.4, 25.5)	
24 weeks	20.5 (10.5, 37.8)	16.3 (10.3, 35.6)	

Abbreviation: LVEF: Left Ventricular Ejection Fraction.

### Secondary endpoints

Blood levels of MPO [baseline: 13.2 (7.9, 24.8), 3 months: 17.7 (11.1, 31.1), 6 months: 19.2 (11.1, 37.8) ng/mL] and Gal-3 [baseline: 6.3 (5.2, 9.6), 3 months: 12.3 (9.8, 16.0), 6 months: 10.3 (8.2, 13.1) ng/mL] increased after ANT chemotherapy, and the longitudinal changes were similar between the placebo and carvedilol groups (*p* for interaction: 0.28 and 0.32, respectively); (Table 4).

**Table 4:** Effect of carvedilol on galectin-3 and myeloperoxidase

	Placebo	Carvedilol	*P* value
	*N* = 148	*N* = 26	
Galectin 3 ng/ml			0.32
Baseline	6.3 (5.2–9.2)	6.4 (5.1, 10.8)	
12 weeks	11.8 (10.2–15.9)	12.9 (9.1, 16.2)	
24 weeks	11.2 (8.4–12.8)	10.3 (7.5–13.1)	
Myeloperoxidase ng/ml			0.28
Baseline	13.3 (7.6–21.0)	13.2 (8.5–29.2)	
12 weeks	15.3 (11.0–25.5)	20.9 (11.2–39.1)	
24 weeks	19.6 (11.9–37.7)	19.2 (10.6–43.7)	

**Figure 2:**
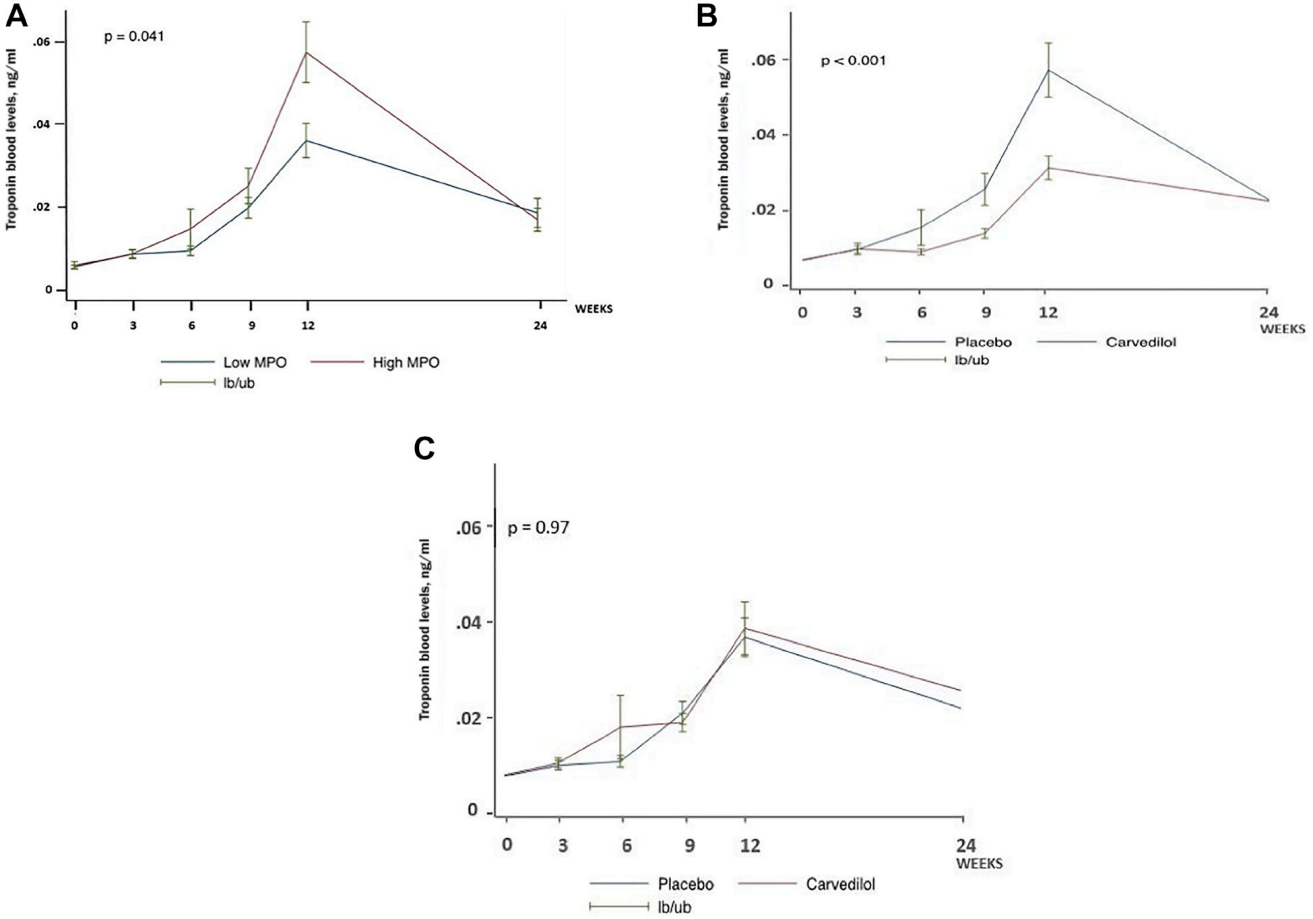
(**A**) TnI blood levels in placebo group according to baseline MPO blood levels. (**B**) Effect of carvedilol on troponin blood levels among women with baseline myeloperoxidase blood levels above median (*n* = 87). (**C**) Effect of carvedilol on troponin blood levels among women with baseline myeloperoxidase blood levels below median (*n* = 87).

In an exploratory analysis, as there is no normal cutoff value established for Gal-3 and MPO in the literature, the MPO and Gal-3 results were splitted in two groups: above and below median. In the placebo group, women with baseline MPO blood levels above median demonstrated a greater increase in TnI blood levels than those with baseline MPO blood levels below median (*p* = 0.041; Figure 2A). Compared with placebo, carvedilol significantly reduced TnI blood levels in women with MPO baseline blood levels above median (*p* < 0.001; Figure 2B), which became similar to the TnI levels in women with baseline MPO blood levels below median (*p* = 0.97; *p* for interaction = 0.009);(Figure 2C). There was no significant interaction between carvedilol treatment and baseline Gal-3 blood levels (*p* for interaction = 0.99).

## DISCUSSION

The main findings of this *post hoc* analysis of the CECCY trial were that blood levels of fibrosis (Gal-3) and oxidative stress (MPO) biomarkers are unrelated to the development of CTX. Also, baseline high MPO blood levels were associated with worse myocardial injury after ANT chemotherapy for breast cancer, as reflected by TnI increase, which was prevented by carvedilol. In addition, MPO blood levels above median identified women who were most likely to benefit from carvedilol for primary prevention.

Regarding the ability of Gal-3 to predict CTX, there are conflicting results. Gal-3 is a beta galactoside binding lectin involved in fibrogenesis and inflammatory response in the failing heart. It is expressed by macrophages and believed to be a mediator of the profibrotic pathway, stimulating cardiac fibroblasts to proliferate and deposit collagen. Pathophysiologically, Gal-3 enhances cardiac fibrosis and influences the immune response, leading to pernicious cardiac remodeling. High levels of this biomarker were associated with left ventricular remodeling and mortality in several studies [15–19], and a previous study demonstrated an association between galectin-3 concentration and future occurrence of clinically manifest heart failure. Concerning the correlation with CTX, a study by Ky et al. in which trastuzumab and doxorubicin were used, no association between CTX and Gal-3 was found, although follow-up only lasted six months [20]. A longer follow-up of fifteen months also failed to demonstrate this association [21], as well as there was no relation with CTX in the analysis of Gal-3 in PRADA trial [8]. In contrast, in the study conducted by Bulten et al., in which only anthracycline was administered, CTX was evaluated with at least 1-year follow-up utilizing ^123^I metaiodobenzylguanidine (^123^I-mIBG) scintigraphy and was related with echocardiography, 2D strain and several biomarkers including Gal-3. Gal-3 was the only biomarker that showed significant correlation with ^123^I-mIBG [16]. Our study did not show any correlation between Gal-3 and CTX and TnI behavior. The reason that might justify this finding is the short-term follow-up, as the study that found correlation had a longer follow-up.

CTX has also been linked to increased MPO values. MPO is a leukocyte-derived enzyme released from activated neutrophils and monocytes, produced due to inflammatory response and oxidative stress [13–22]. Increase in MPO blood levels was related with cardiovascular events in patients presenting with chest pain [23], as well as with the presence of heart failure in the general population [24, 25]. Also, it has a prognostic value upon right ventricular systolic dysfunction [26] and incremental prognostic information in patients with acute heart failure [13]. Ky et al. described an additive value when MPO is associated to TnI, enhancing prediction of subsequent CTX in breast cancer patients treated with doxorubicin and trastuzumab [20]. MPO remained a predictor of CTX risk over the entire course of the aforementioned chemotherapy [21]. In our study, although MPO was not related to decrease in LVEF, women with higher MPO baseline values had greater myocardial injury and were most likely to benefit from carvedilol. A possible explanation is that, as in another studies mentioned before, MPO may have an additive value when used with TnI by identifying patients with greater risk of myocardial injury. As inflammation enhances, more oxidative stress occurs and consequently these patients achieve a better response to carvedilol.

Anthracyclines interfere with the replication of rapidly proliferating cancer cells through several modes of action. Doxorubicin reduces mitochondrial NADH dehydrogenase to form a semiquinone radical that participates in further redox cycling [27]. The resulting hydrogen peroxide may be converted to toxic free radicals in the presence of intracelular iron. Furthermore, doxorubicin forms complexes directly with iron to generate reactive oxygen species [28]. By intercalating with DNA base pairs and stabilizing the Topoisomerase (Top) 2α complex after DNA cleavage, they increase DNA breaks and prevent DNA and RNA synthesis [29]. Mechanistic understanding of anthracycline cardiomyocyte injury has focused on building reactive oxygen species (ROS) and targeting Top 2α in cardiomyocytes. Iron accumulation within the mitochondria is associated with futile redox cycling and increased ROS production [30]. ROS production is harmful but it was uncertain to what degree it was a cause or consequence of anthracycline –mediated cell injury [31]. The detrimental effects of ANT might be also mediated by deleterious effects of doxorubicin on endothelial cells. Increased permeability of the endothelium prolongs cardiomyocytes exposition to doxorubicin, which directly damages the myocytes. ROS disrupts endothelium-based cardiac myocyte supportive functions and promotes pathological release of endothelial cell-derived endothelin-1 (ET-1), prostaglandin I2 (PGI2), nitric oxide (NO), and endothelium-derived neuregulin-1 (NRG-1). This biochemical imbalance reduces the survivability and adaptability of cardiac myocytes [11]. Translational studies have focused on identifying new markers of oxidative stress in the setting of ANT cardiotoxicity [28]. The underlying mechanism of ANT injury helps to clarify our results, bringing a biomarker of oxidative stress as a predictor of myocardial injury, acting together with TnI.

Although beta-blockers appear to prevent doxorubicin-cardiotoxicity according to preclinical data and small clinical trials [32–37], this has not been confirmed in larger recent trials [7, 9]. The recent CECCY trial has shown that carvedilol attenuated the increase of Tn blood levels, but it did not reduce the risk of ANT-CTX, as defined by a decrease in LVEF. A possible explanation for these findings may be a low prevalence of CTX in the recent era with the contemporary doses of ANT and a low prevalence of cardiovascular comorbidities. Moreover, the association between TnI increase and subsequent LVEF reduction has been controversial, particularly in recent large trials [5–9, 36–38]. It is conceivable that a slight increase in TnI blood levels during chemotherapy may reflect unexpressive myocardial injury, which might not result in myocardial dysfunction.

Considering the exposed evidence, our study contributes to reaffirm the oxidative stress as an important mechanism of ANT CTX and ratifies the role of MPO as a prognostic biomarker, enhancing the TnI predictor power. These findings were similar to previous studies [20, 21] that also evaluated the role of MPO in this context. However, this was the only study in which only ANT was administered, highlighting the correlation between ANT use, MPO behavior and myocardial injury. Besides that, the anti-oxidant effects of carvedilol could justify the protective effects in myocardial injury. The correlation of high MPO with greater response to carvedilol will help to select the more CTX vulnerable patients and perhaps helps carvedilol to obtain a positive effect in CTX prevention.

### Study limitations

Our study has some limitations that deserve attention. First, we lost 18 patients from the original trial due to hemolyzed blood samples. Second, it was a post hoc analysis. Third, our main result is based on a random division of biomarker values between above and below median. Still, there are no reference cutoff values for MPO or Gal-3 and we performed a similar division from other studies using these biomarkers [12, 20, 21]. Fourth, theoretically being more inflamed, patients with breast cancer disease would have a greater value of baseline MPO. However, in our study, the MPO baseline levels were lower than the median of subjects from a healthy population [39]. However, unlike other studies, which performed one baseline measurement, we did at least two-blood sample analysis in three different periods. Fifth, because the incidence of CTX was low in the CECCY trial, our analysis may have been underpowered to detect an association between biomarkers of oxidative stress and CTX. On the other hand, our analysis was derived from the largest prospective randomized double blind trial evaluating the use of cardiovascular drugs for primary prevention of ANT CTX.

## MATERIALS AND METHODS

### Study design

A post hoc analysis was performed in patients who attended CECCY trial [9]. 174 women had her blood samples stored during the study and an analysis of MPO and Gal-3 was performed. CECCY trial was a prospective, double blind, randomized, placebo-controlled study, conducted in the Heart Failure Department of Heart Institute (INCOR) and the Cancer Institute, São Paulo, Brazil. Patients were referred from the Cancer Institute and the Heart Failure Team of the Heart Institute was responsible for allocation, randomization, and adjustment of the dose of carvedilol/placebo. Data were collected, managed, and analyzed by the Heart Failure Team after the end of the study. The institutional review board at both institutions approved the trial protocol. All participants were informed about the research objectives, research protocol and treatment alternatives involved in the study. All participants provided written informed consent to participate in the study. The trial was registered at https://clinicaltrials.gov (NCT 01724450) before study initiation.

### Study patients

Eligibility requirements and exclusion criteria from the CECCY trial were described elsewhere [9]. Briefly, it included all consecutive patients with HER2-negative breast cancer tumor status and therapy that included ANT, cyclophosphamide and taxane from April 23, 2013 to January 3, 2017. The standard chemotherapy protocol comprised 4 cycles of cyclophosphamide 600 mg/m^2^ and doxorubicin 60mg/m^2^ every 21 days (with a total cumulative dose of 240 mg/m^2^), followed by paclitaxel 80 mg/m^2^ weekly for 8 weeks.

### Randomization, allocation and intervention

Randomization, allocation and intervention were described earlier in the CECCY trial [9]. In summary, randomization was on a 1:1 ratio to receive carvedilol or placebo. The randomization included a predefined stratification according to menopause status, considering the potential difference in CTX risk between pre and post-menopause [40].

Carvedilol and placebo were administered in a progressive manner with incremental dosing at 3-week intervals to a maximum dose of 25 mg every 12 h or until the appearance of intolerable symptoms or heart rate ≤60 beats/min or systolic blood pressure <110 mmHg. Carvedilol and placebo were continued until completion of chemotherapy.

### Study procedures

All eligible patients underwent a baseline transthoracic echocardiogram and routine laboratory tests including biomarkers before randomization. Troponin I (TnI) and brain natriuretic peptide (BNP) were measured at the same time of sample collection but blood samples was stored for posterior analysis. If the patient met the eligibility criteria, the randomization was performed. After randomization, the medication was initiated on the first day of chemotherapy. The following sequential measurements of biomarkers were performed in a median of 19 days after each ANT cycle. Venous blood samples were collected in standard tubes for plasma with both EDTA and heparin, processed at 3100 rpm for 15 minutes, divided into aliquots and stored at −80°C until the time of assay in a single batch. Stored blood samples were obtained at baseline, 3 and 6 months after the beginning of randomization.

### Laboratory analysis

The quantitative TnI determination was obtained by means of a 3-step sandwich immunoassay using direct chemiluminescent technology and constant amounts of 2 monoclonal antibodies. An auxiliary reagent was included to reduce nonspecific binding using the ADVIA Centaur TnI-Ultra commercial kit (Siemens Healthcare Diagnostics, Tarrytown, New York). The level of detection was 0.006 ng/ml. Levels <0.006 were reported as 0.005 ng/ml. The normal range of TnI was <0.04 ng/ml.

MPO and Gal-3 were measured using Luminex xMAP technology through MILLIPLEX MAP KIT (Merck Laboratories). Plasma specimens were prepared for analysis in a 96-well plate utilizing a Milliplex Map Human Circulating Cancer Biomarker Magnetic Bead Panel 3 (Millipore Corp., Billerica, MA, EUA), following the kit-specific protocols provided by Millipore. Analytes were quantified using a Magpix analytical test instrument, which utilizes xMAP technology, multiple analyte profiling (Luminex Corp., Austin, TX, USA), and xPONENT 4.2 software (Luminex). xMAP technology uses fluorescente-coded color magnetic microspheres coated with analyte-specific capture antibodies to simultaneously measure multiple analytes in a single specimen. After micro-spheres have captured the analytes, a biotinylated detection antibody binds to that complex. Streptavidin PE then attaches as a reporter molecule. Inside the instrument, magnetic beads are held in a monolayer by a magnet, where two LEDs are used to excite the internal micro-sphere dye and the dye of the reporter molecule, respectively. A CCD camera captures these images, which are then analyzed by Milliplex Analyst software (Millipore). Biomarkers concentrations (ng/ml) were determined on the basis of the fit of a standard curve for mean fluorescence intensity versus ng/ml.

### Echocardiography

Transthoracic echocardiography was performed with a commercially available system (Envisor Philips, Philips Healthcare, Andover, Massachusetts). All the measurements were performed and reported according to the recommendations of the American Society of Echocardiography [41]. LVEF was measured by Simpson rule, throughout apical 4-and-2-chamber views. We also assessed the following echocardiographic parameters: left atrium diameter, interventricular septum diameter, posterior wall thickness, LV end-diastolic diameter, LV end-systolic diameter and mitral inflow with the use of Doppler echocardiography [42]. These images were stored on a secure institutional drive. All scans were read by experienced board-certified echo cardiographers who were blinded to all clinical characteristics.

### Study endpoints

In this *post hoc* analysis, the primary endpoint was the correlation between the biomarkers Gal-3 and MPO with CTX, as defined by a drop in LVEF of at least 10% from baseline at any point until the end of chemotherapy at 6 months [43]. The secondary endpoint is an exploratory analysis of the longitudinal changes of MPO and Gal-3 and their relation with TnI.

### Statistical analysis

Levels of galectin-3 and MPO were not normally distributed and were presented as median (25th percentile, 75th percentile). Because of their right skewed distribution, the levels of galectin-3 and MPO were log transformed for the analysis.

We performed longitudinal mixed-effect models to compare the trajectories of these biomarkers over time between groups. As originally reported in the CECCY trial, the log-transformed troponin blood levels had a biphasic trajectory over time [9]. Consistently with the main trial, we included time in the model as a quadratic function (time and time squared) comparing the trajectories of log-transformed troponin blood levels between groups. Additionally, we tested for nonlinearity of the trajectories of log-transformed galectin-3 and log-transformed MPO by comparing a quadratic model – including time squared – with a linear model using a likelihood ratio test, and we assumed a linear behavior if a *p* value was >= 0.05. The trajectories were compared between groups using a likelihood ratio test between a model with and without group-time and group-time squared interaction terms.

Finally, we evaluated whether the trajectories of log-transformed troponin blood levels differed according to blood levels of MPO at baseline, categorized as above or below median. Because of the known effect of carvedilol on troponin blood levels, this analysis was performed separately in the placebo and carvedilol treatment groups. We tested whether the magnitude of the effect of carvedilol differed according to baseline MPO blood levels by performing a likelihood ratio test between a model with and without treatment-MPO-time and treatment-MPO-time squared interaction terms. The same procedure was performed to compare trajectories of log-transformed troponin blood levels according to blood levels of galetin-3. The statistical analysis was performed using Stata version 15.0 (StataCorp, College Station, TX, USA) and the level of significance was defined as *p* < 0.05.

### Clinical perspectives

As the isolated Tn elevation after ANT chemotherapy failed to correlate with a further development of systolic disfunction, MPO blood levels contributes to identify patients who are more likely to develop cardiotoxicity. A better stratification of patients could identify those who would really benefit from primary prevention with carvedilol.

### Translational outlook

As biomarkers are the most premature identifiable sign of myocardial injury after ANT chemotherapy, and as so far no biomarker alone has been able to predict LVEF dysfunction, the combination of multiple biomarkers could increase sensitivity and accuracy in identifying ANT cardiotoxicity. In this study MPO has been shown to increase troponin sensitivity and helped to identify those who would benefit most from carvedilol as primary prevention. Future research on the association between MPO and Tn is needed, as well as the identification of other biomarkers.

## CONCLUSIONS

In this subanalysis of the CECCY trial, we concluded that carvedilol did not change galectin-3 and MPO blood levels after ANT chemotherapy in women with breast cancer. Although these biomarkers did not predict the development of CTX, MPO baseline blood levels above median was associated with worse myocardial injury and identified women who were more likely to benefit from carvedilol for primary prevention.
